# The complete mitochondrial genome of marmoset rats *Hapalomys delacouri* (Rodentia: Muridae)

**DOI:** 10.1080/23802359.2019.1627938

**Published:** 2019-07-12

**Authors:** Bin Zhang, Xuelong Jiang

**Affiliations:** a Department of Anatomy and Neurobiology, Biology Postdoctoral Workstation, School of Basic Medical Sciences, Central South University, Changsha, China;; b State Key Laboratory of Genetic Resources and Evolution, Kunming Institute of Zoology, Chinese Academy of Sciences, Kunming, 650223, China

**Keywords:** *Hapalomys delacouri*, mitochondrial genome, Muridae

## Abstract

The marmoset rats (*Hapalomys delacouri*) is a rarely investigated rodent. It occupies important ecological niche and phylogenetic position. The complete mitochondrial genome of *H. delacouri* was determined for the first time by long PCR and second-generation sequencing. The results showed that the entire mitogenome of *H. delacouri* was 16,316 bp long (deposited in GenBank with accession number MK850463). The gene order and orientation were the same as the most rodent. Only tRNA^Lys^ (TTT) and tRNA^Ser^ (AGN) of 22 transfer RNAs showed atypical cloverleaf structures, lacking a dihydrouridine (DHU) arm. The newly added *H. delacouri* mitogenome will benefit population genetic and phylogenetic studies in the rodent.

The genus *Hapalomys* (Blyth 1859), commonly named as marmoset rat, is a rarely investigated group rodent for their unique habitat, patchy distribution, individual rarity, and specimen scarcity (Musser and Carleton 2005; Arber et al. 2017). The first species of this genus was described in 1859 based on specimens from south Burma (Blyth 1859). Until One century later, reviewed this taxon and only recognized two valid species (*Hapalomys longicaudatus* and *Hapalomys delacouri*) with two subspecies (*Hapalomys d. pasqueri* and *Hapalomys d. marmosa*) based on tens of individuals (Musser 1972). This mitochondrial genome will provide comparable information for understanding the mitochondrial evolution and animal physiology.we are so lucky to gain one specimen that identified as *H. delacouri* from Shiwandashan National Forest Park, Shangsi, Guangxi Zhuang Autonomous Region, China (21.9075° N, 107.91° E) in the field collection. Voucher material has been deposited at the Kunming Institute of Zoology, China.

Total genomic DNA was extracted using sodium dodecyl sulfate method (Sambrook and Russell 2001). The mitochondrial genome was amplificated, sequenced and assembled following Chen et al. (Chen et al. 2016). The assembled Mitochondrial genome was annotated using the web serve of MITOS (Bernt et al. 2013) with default parameters. thirteen PCGs were compared with other murine using MEGA 7.0 (Kumar et al. 2016). Transfer RNA genes were scanned and predicted secondary structure with tRNAscanSE1.21 (Lowe and Chan 2016) (http://lowelab.ucsc.edu/tRNAscanSE/).

The mitogenome of *H. delacouri* is a closed-circular DNA molecule of 16,316 bp in length (accession number MK850463), containing 13 PCGs (*ATP6*, *ATP8*, *COXI*–*III*, *NAD1*–*6*, *NAD4L*, *CYTB*), 2 rRNAs genes (srRNA and lrRNA), and 22 tRNAs genes and the putative control region. The order and length of genes are similar to that of known murine species. Genes of the eight tRNA (*Gln*, *Ala*, *Asn*, *Cys*, *Tyr*, *Ser*, *Glu*, and *Pro*) and *ND6* are located on the light strand identical to find in other vertebrates.

The mitochondrial genome of *H. delacouri* includes 13 PCGs, totally 11,400 bp long, accounting for 69.87% of the whole length and encoding 3789 amino acids. The A + T content of 13 PCGs was 72.35%.

Twenty-two tRNA genes interspersed throughout the 13 PCGs region, transferring 20 specific amino acid (two for leucine and two for serine), length from 59 to 75 bp. The second structure of 20 tRNAs were predicted as typical cloverleaf secondary structures, except tRNALys (TTT) and tRNASer (AGN). The unusual tRNALys (TTT) lacked a D ring and tRNASer lacked dihydrouridine (DHU) arm, which was replaced by a simple loop.

To further validate its phylogenetic position, nine complete mitochondrial genome sequences of other Murinae and *Gerbilliscus leucogaster* (as outgroup) were downloaded from GenBank to construct a neighbor-joining tree (Saitou and Nei 1987) using MEGA7 (Kumar et al. 2016) with 1000 bootstrap replicates. MAFFT (Katoh and Standley 2013) was used to the aligned data matrix. Our tree showed that *H. delacouri* is first split from Murinae with over 100% bootstrap support (Figure 1). The phylogenetic relationships of other species are in accord with the previous study (Aghova et al. 2018).

**Figure 1.**
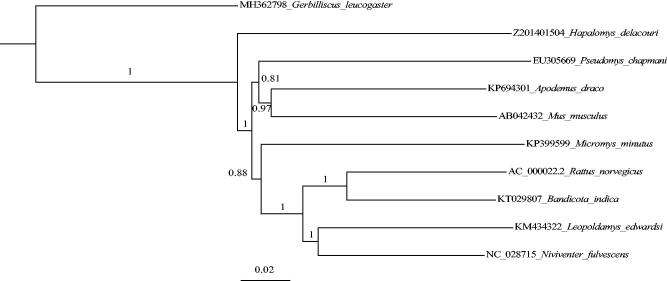
Maximum-likelihood tree of *Hapalomys delacouri* and other Murinae species based on complete mitogenome with 1000 bootstrap replicates. Genbank accession number for other sequences is listed ahead species name.
